# Supplementary material for: Imaging the transient heat generation of individual nanostructures with a mechanoresponsive polymer
Source: Nat Commun. 2017 Nov 14;8:1498. doi: 10.1038/s41467-017-01614-0 (PMC5686141; doi:10.1038/s41467-017-01614-0)
Supplement: Supplementary file 1 — Supplementary Information [file 41467_2017_1614_MOESM1_ESM.pdf]

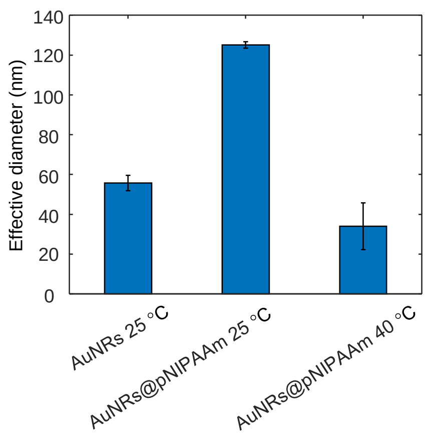

Supplementary Figure 1. Dynamic light scattering hydrated radius response to solution temperature change. Effective diameter of AuNRs, AuNRs@pNIPAAm at 25 °C and AuNRs@pNIPAAm at 40 °C. Error bars, ±1 s.d. with *n*=3 each.

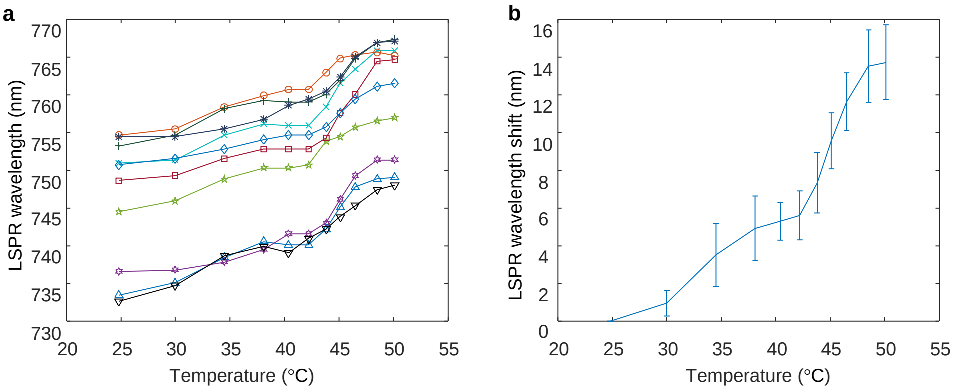

Supplementary Figure 2. Thermal response of AuNRs@pNIPAAm-*co*-pAAm. (**a)** Temperature-dependent scattering spectra peak wavelength of ten gold nanorods modified by pNIPAAm-*co*-pAAm with LCST at 39 °C. **(b)** Mean scattering spectral redshifts of the ten particles as a function of temperature. Error bars, ±1 s.d.

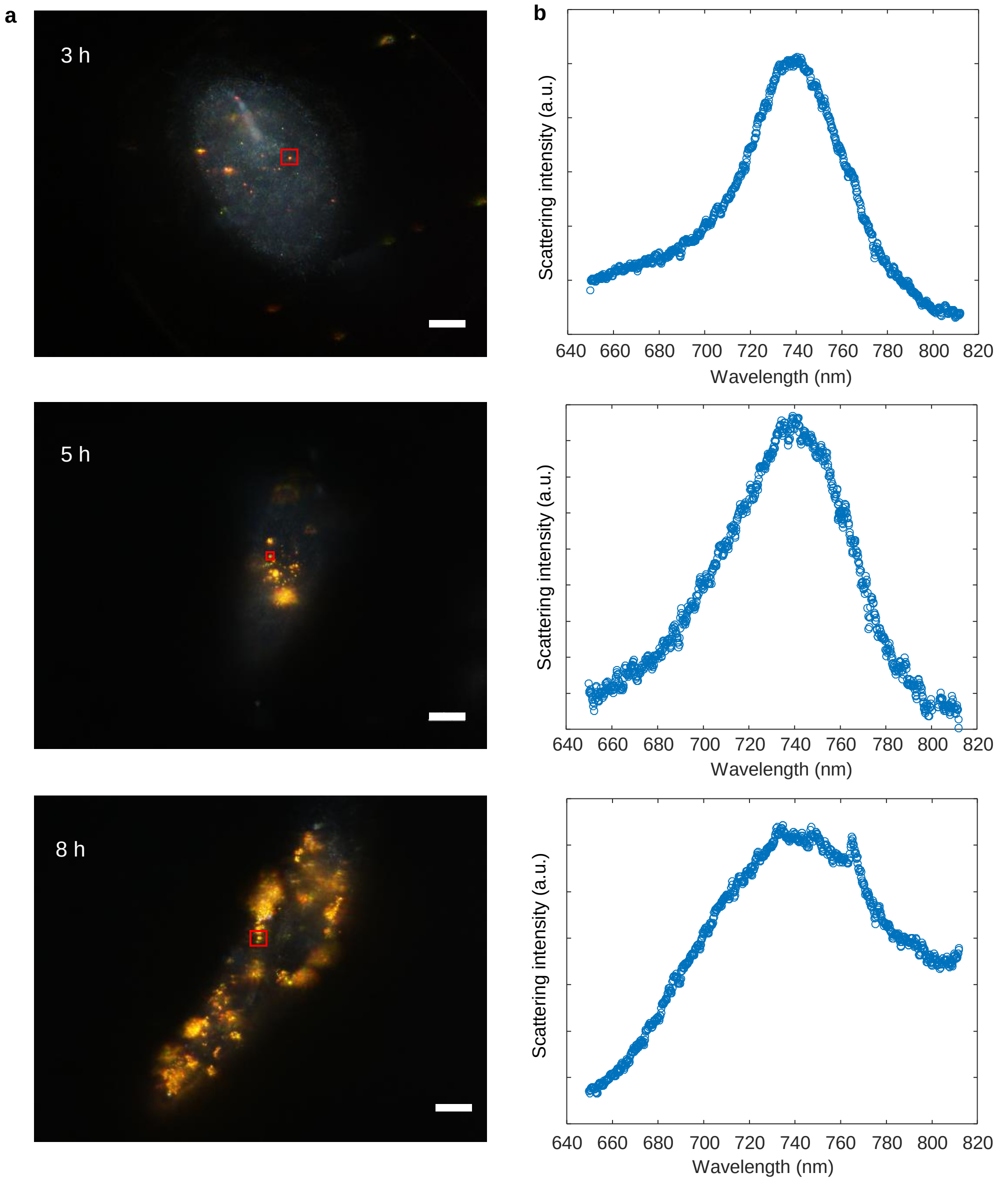

Supplementary Figure 3. **Optimization of intracellular probes density. (a)** Broadband dark-field images of HeLa cells incubated with 40 pM AuNRs@pNIPAAm/RGD for 3, 5 and 8 h, respectively. **(b)** Scattering spectra of the dots labeled by squares in corresponding images. Scale bar is 10 μm.

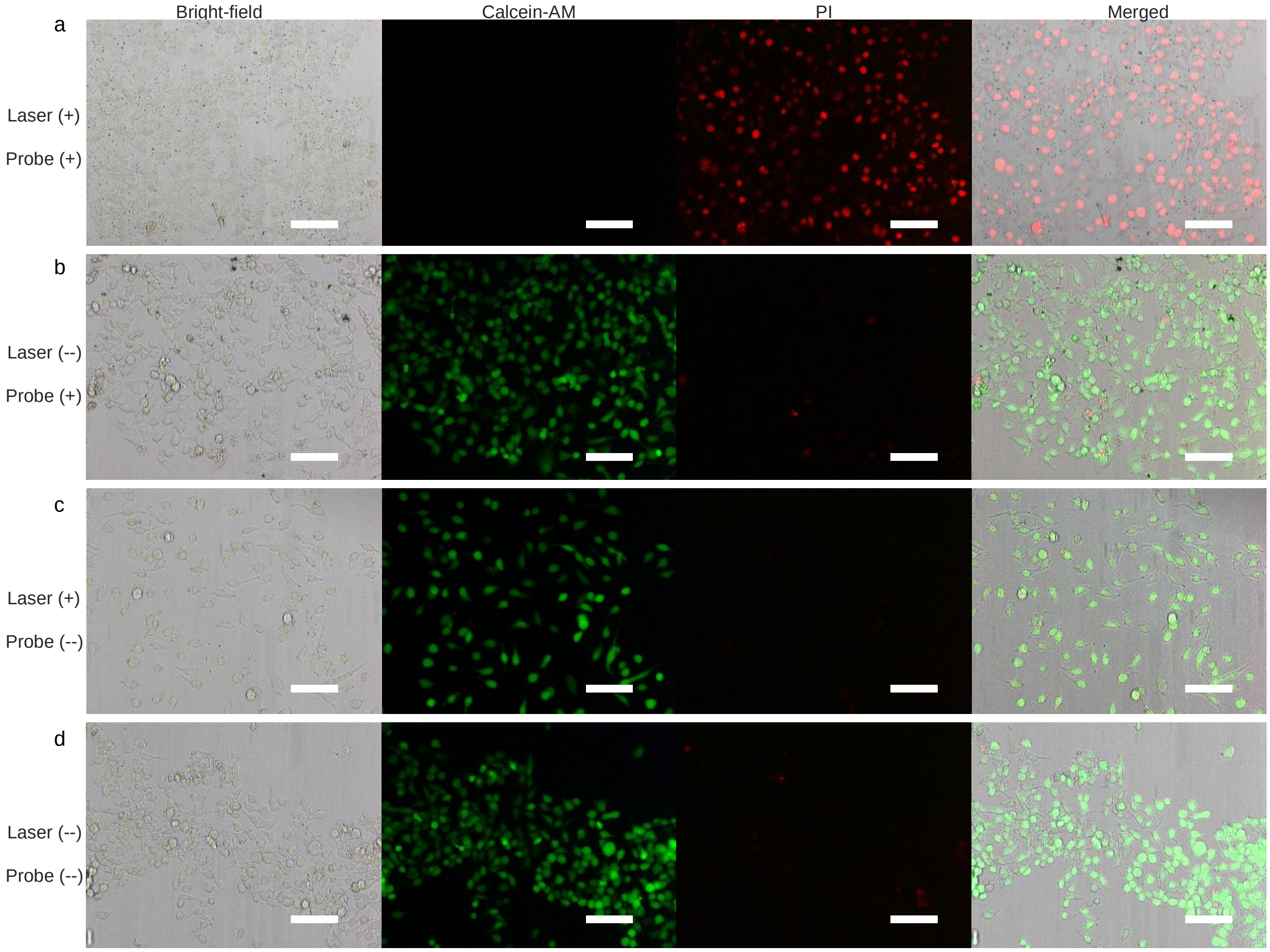

Supplementary Figure 4. Performance of AuNRs@pNIPAAm@RGD in Photothermal therapy. (a) The bright-field image, Calcein-AM fluorescent image, Propidium Iodide (PI) fluorescent image and the merged images of HeLa cells incubated with 40 pM AuNRs@pNIPAAm@RGD and then with 10 min laser irradiation. **(b)** The same images of HeLa cells incubated with 40 pM AuNRs@pNIPAAm@RGD. **(c)** The same images of HeLa cells with 10 min laser irradiation. **(d)** The same images of untreated HeLa cells. Scale bar is 100 μm.

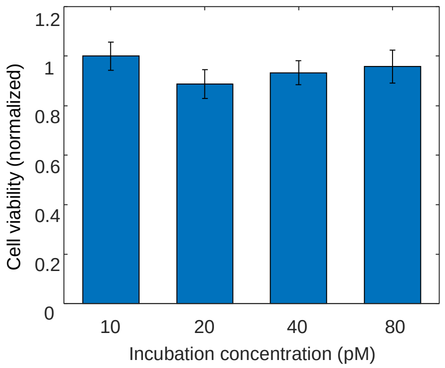

Supplementary Figure 5. Cytotoxicity assay of AuNRs@pNIPAAm@RGD. Viability of HeLa cells incubated with 0, 20, 40 and 80 pM AuNRs@pNIPAAm@RGD for 48 h. Error bars, ±1 s.d. with *n*= 5 each.

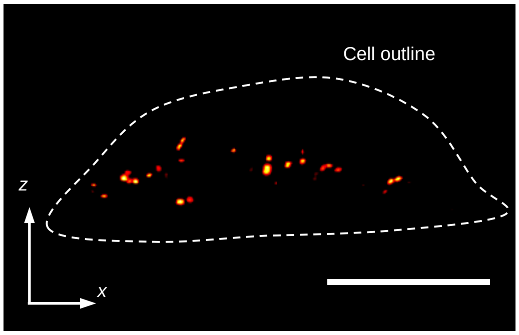

Supplementary Figure 6. Three-dimensional dark-field image of intracellular AuNRs@pNIPAAm/RGD (dash line indicated the cell outline) reconstructed from serial *z*-scanned images. Scale bar is 10 μm.

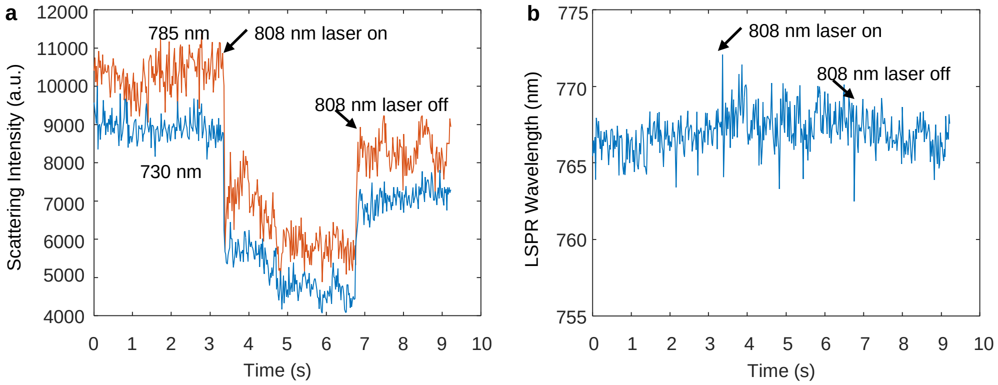

Supplementary Figure 7. Illustration of the light-induced rotation of AuNRs@pNIPAAm/RGD particles. (a) Time-dependent scattering intensity of a single AuNRs@pNIPAAm/RGD particle in 785 (up) and 730 nm (down) channel, respectively. **(b)** Converted time-dependent LSPR wavelength from the data in **(a)**.

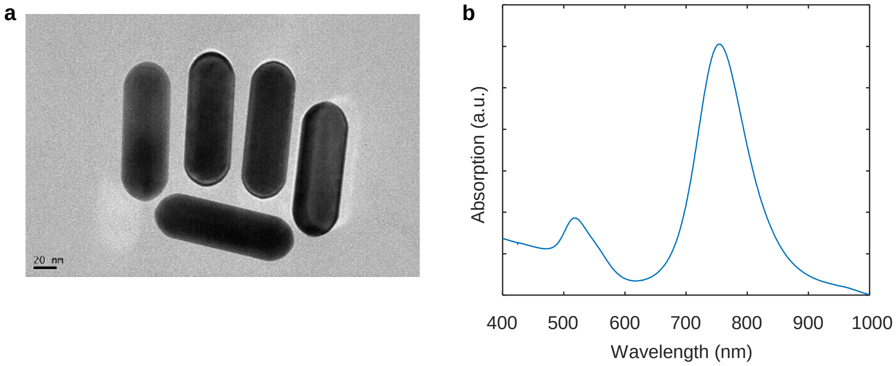

Supplementary Figure 8. Characterization of the bare gold nanorods. TEM image (a) and UV-vis spectra (b) of the bare gold nanorods. Scale bar is 20 nm.

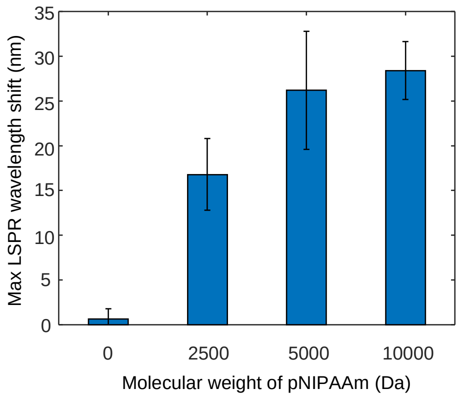

Supplementary Figure 9. Optimization of pNIPAAm synthesis. Maximum temperature-induced LSPR wavelength shifts of bare gold nanorods, AuNRs@pNIPAAm assembled with 2500 Da, 5000 Da and 10000 Da pNIPAAm. Error bars, ±1 s.d. with *n*= 20 each.

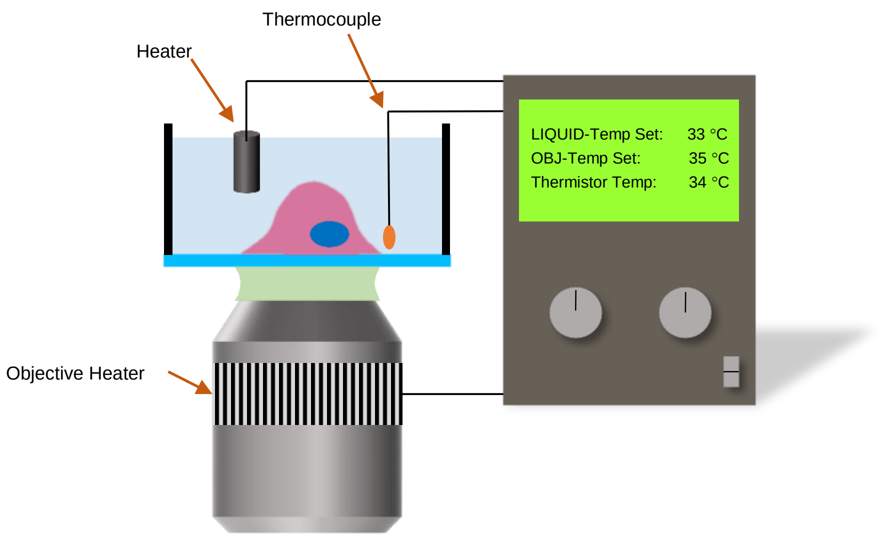

Supplementary Figure 10. Setup of a homemade microscope-compatible incubator. The media temperature is controlled by two heaters for objective and media respectively. A thermocouple is attached onto the cover slide to measure the temperature near the cell. Heat power of two heaters are controlled by the measured temperature to keep a stable environment for following experiments.
