# Supplementary material for: Imaging the transient heat generation of individual nanostructures with a mechanoresponsive polymer
Source: Nat Commun. 2017 Nov 14;8:1498. doi: 10.1038/s41467-017-01614-0 (PMC5686141; doi:10.1038/s41467-017-01614-0)
Supplement: Supplementary file 3 — Description of Additional Supplementary Files [file 41467_2017_1614_MOESM3_ESM.pdf]

Description of Supplementary Files

File Name: Supplementary Movie 1
Description: **Movie demonstrating the 730 nm-channel snapshots when samples rotate from 0 to 360°.** Scattering images of gold nanorods illuminated by the 730-nm laser are recorded in the interval of 15°, and the red double arrow indicates the polarization direction.

File Name: Supplementary Movie 2
Description: **Movie demonstrating the 785 nm-channel snapshots when samples rotate from 0 to 360°.** Scattering images of gold nanorods illuminated by the 785-nm laser are recorded in the interval of 15°, and the red double arrow indicates the polarization direction.

File Name: Supplementary Movie 3
Description: **Movie demonstrating the spectra mapping converted from two-channel snapshots when samples rotate from 0 to 360°.** The convert process is based on equation (2), and the wavelength is not affected by the direction variation.

File Name: Supplementary Movie 4
Description: **Movie demonstrating the three-dimension dark-field snapshots reconstructed from serial images when samples rotate from 0 to 360°.** The reconstructed three-dimension movie demonstrates that plenty of AuNRs@pNIPAAm/RGD are evenly dispersed inside the cell.
